# Emergence of antimicrobial resistance to *Pseudomonas aeruginosa* in the intensive care unit: association with the duration of antibiotic exposure and mode of administration

**DOI:** 10.1186/s13613-017-0296-z

**Published:** 2017-06-29

**Authors:** Erlangga Yusuf, Bruno Van Herendael, Walter Verbrugghe, Margareta Ieven, Emiel Goovaerts, Kristof Bergs, Kristien Wouters, Philippe G. Jorens, Herman Goossens

**Affiliations:** 1 Department of Microbiology, Antwerp University Hospital (UZA), University of Antwerp, Wilrijkstraat 10, 2650 Edegem, Belgium; 2 Department of Intensive Care Medicine, Antwerp University Hospital (UZA), University of Antwerp, Wilrijkstraat 10, 2650 Edegem, Belgium; 3 Department of Hospital Hygiene and Infection Control, Antwerp University Hospital (UZA), University of Antwerp, Wilrijkstraat 10, 2650 Edegem, Belgium; 4 Department of Biostatistics, Antwerp University Hospital (UZA), University of Antwerp, Wilrijkstraat 10, 2650 Edegem, Belgium; 5 GZA Hospitals, Antwerp, Belgium

**Keywords:** *Pseudomonas aeruginosa*, Antibiotic resistance, Extended infusion

## Abstract

**Background:**

Antibiotics are frequently used in intensive care units (ICUs), and their use is associated with the emergence of bacterial resistance to antibiotics. The aim of this study was to investigate the association between the emergence of *Pseudomonas aeruginosa* resistance and the duration of antibiotic exposure or mode of administration in an ICU unit.

**Methods:**

A 4-year cohort study of intensive care unit was performed in patients with *P. aeruginosa* isolates from clinical specimens, initially susceptible to the investigated antibiotics (piperacillin/tazobactam, ceftazidime, ciprofloxacin, meropenem and amikacin). Odds ratios (ORs) with 95% confidence interval (95% CI) of emergence of resistance were calculated using logistic regression analysis for various exposure periods to antibiotics (1–3, 4–7, 8–15 and >15 days) relative to no exposure with adjustment for age, sex, Simplified Acute Physiology Score 3 (SAPS 3) and length of stay. ORs on the emergence of *P. aeruginosa* resistance were also calculated for the various modes of administration.

**Results:**

Included were 187 patients [mean age 61 years, 69% male, mean SAPS 3 score (SD): 59 (12.3)]. None of the antibiotics investigated showed the emergence of resistance within 1–3 days. Significant meropenem resistance emerged within 8–15 days [OR 79.1 (14.9–421.0)] after antibiotic exposure unlike other antibiotics (>15 days). No difference was observed between intermittent and extended administration of meropenem and between beta-lactam mono- or combined therapy.

**Conclusions:**

Use of meropenem was associated with the emergence of resistance as soon as 8 days after exposure to the antibiotic.

## Background

*Pseudomonas aeruginosa* is responsible for 9% of all healthcare-associated infections [1], and the resistance to *P. aeruginosa* is increasing [2]. Data from the European Centre for Disease Prevention and Control show that around 18% of *P. aeruginosa* strains are resistant to carbapenems, 15% resistant to at least three out of five antimicrobial classes with an antipseudomonal spectrum (piperacillin/tazobactam, ceftazidime, fluoroquinolones, carbapenems and aminoglycosides) and even 5% resistant to all five [3]. Antimicrobial resistance is of particular importance in the intensive care unit (ICU) because antibiotic use in this setting is extremely common [4]. Several studies have explored the risk factors for *P. aeruginosa* antimicrobial resistance in non-critically ill patients [5, 6]. These studies showed that among antipseudomonas antibiotics, meropenem was associated with the highest risk of resistance emergence [4, 7]. The risk factors for emerging antimicrobial-resistant *Pseudomonas aeruginosa* in the ICU are also worth investigating, especially to answer the question how quick the antibiotic resistance emerges after the exposure to antibiotics. Also, worthy of investigation is whether the use of extended infusion of meropenem and the combination of beta-lactam antibiotics with aminoglycosides are associated with less risk of the emergence of resistance. Extended infusion of meropenem (over 3–4 h) has been shown to improve microbiologic and clinical cure since it shows a time-dependent effect on bacterial eradication [8] but it is not clear whether extended infusion also reduces the emergence of resistance. Combination of beta-lactam antibiotics with aminoglycosides is often used in daily practice [9], for example in critically ill patients, because it increases the chance of treating the patients with one active antibiotic. Arguably, the risk of inappropriate antibiotic therapy (no active molecule) will increase in case of use of a beta-lactam alone, and inappropriate therapy is usually associated with a poor prognosis. Yet, the use of combination has not shown to have an added benefit, for example in neutropenic patients [10].

Therefore, the aim of this study was twofold: first to quantify the duration of antibiotic exposure and other risk factors for emergence of resistance and second to investigate whether the mode of administration of meropenem and combination beta-lactams and aminoglycosides were associated with lower emergence of resistance of previously susceptible *P. aeruginosa*.

## Methods

### Study setting and population

The cohort study was performed between June 2007 and June 2011 in adult patients admitted to the ICU of the Antwerp University Hospital (UZA) for at least 48 h. This ICU consists of 45 beds with approximately 2600 adult admissions yearly; it serves as tertiary care hospital in a city with the population of around 500,000 inhabitants. Included in the study were patients with >1 clinical (i.e., not screening) culture positive with *P. aeruginosa* from any site of the body (blood, upper (sputum) and lower respiratory tract (bronchoalveolar lavage), and wound, that were taken from a patient at least 2 days apart. Patients who were re-admitted after an initial stay in the ICU during the observed period as well as those on antibiotics prior to admission were excluded. On the included patients, we collected demographic data (age and sex), clinical characteristics (reason of ICU admission and Simplified Acute Physiology Score 3 ((SAPS 3), a validated marker of disease severity [11]), use of mechanical ventilation) and data on all antimicrobial drugs the patients received during the ICU stay (duration and mode of administration). The patients received standard dose of antibiotics or adjusted dose according to renal clearance. The standard intravenous doses were 4 g/2 g q.i.d. for piperacillin/tazobactam, 6 g o.d. for ceftazidime, 400 mg b.d. for ciprofloxacin, 1 g t.d. for meropenem and 15 mg/kg divided into three doses for amikacin. Amikacin was, apart from treatment of endocarditis with gentamicin, the only aminoglycoside used in our ICU.

### Specimens

All cultures were analyzed according to standard hospital and laboratory protocol. Antibiotic susceptibility testing was performed using disk diffusion technique on Mueller–Hinton agar for piperacillin/tazobactam, ceftazidime, ciprofloxacin, meropenem and amikacin [12]. Zone diameters were interpreted according to CLSI (Clinical & Laboratory Standards Institute) guidelines (CLSI M100) as susceptible, intermediate or resistant. Intermediate isolates were considered in this present study as susceptible since the intermediate category also implies that a higher-than-normal dosage of a drug can be used [13].

### Statistical analysis

All variables were assessed and described according to their distribution. Normally distributed variables were presented as mean with standard deviation (SD), and not normally distributed variables as median with range.

The outcome of the investigation was the emergence of resistance in *P. aeruginosa*, defined as phenotype switch of the relevant antibiotic from susceptible/intermediate at the initial culture to resistant at any follow-up culture. The determinant variable was the duration of the relevant antibiotic used by the patients. The patients were divided into groups according to the number of days of antibiotic exposure according to the literature [6, 14] as follows: not exposed, 1–3, 4–7, 8–15 and >15 days.

Goodman and Kruskal’s gamma tests were first used to investigate an association between higher categories of exposure and higher numbers of the emergence of resistance. Associations were considered significant if p-values were <0.05. To further quantify the effect of antibiotic exposure on emergence of resistance per antibiotic, logistic regression analysis was performed to calculate odds ratios (ORs) with 95% confidence interval (95% CI) of emerging resistance to an antibiotic for patients in one of the exposed groups (1–3, 4–7, 8–15 and >15 days) compared to the group without exposure with adjustment for possible confounders age, sex and SAPS 3 scores. Further adjustment was made for the length of stay. The analyses were repeated using the emergence of resistance antibiotic on the phenotype switch of any antipseudomonal antibiotic as the outcome. Re-analyses were also performed after excluding possible cross-transmission. Arbitrarily, we defined possible cross-transmission as cases with phenotype switch to identical antibiogram with a potential source patient with overlapping time periods of 30 days. Due to the small number of the included study participants, the effect of interaction between antipseudomonal antibiotics was not investigated.

To investigate the effect of meropenem administration through extended or intermittent infusion and the effect of combination therapy versus monotherapy, the ORs (95% CI) were calculated using logistic regression analysis comparing the risk of emerging resistance in one regimen versus the risk in the comparator regimen. All analyses were performed using IBM SPSS Statistics for Windows version 23.0 (IBM Corp., Armonk, NY).

## Results

### Demographic of study participants

**Fig. 1:**
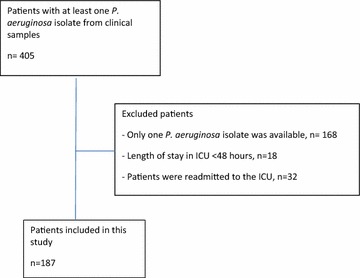
Flowchart of included patients

We identified 405 patients with at least one positive culture with *P. aeruginosa* during the study period. After exclusion of patients with only one isolate (*n* = 168), the length of stay <48 h (*n* = 18) and patients re-admitted to the ICU (*n* = 32), 187 patients (mean age of 61 years, 69% male) were included. The flowchart of included patients is presented in Fig. 1. The included patients had mean SAPS 3 score of 59 (12.3). Most of the patients had mechanical ventilation (84.0%) and vasopressors (88.8%) (Table 1).

**Table 1:** Demographic and clinical characteristics of the study cohort (*n* = 187)

Characteristics	Values
Mean age (SD), years	61 (14)
Male gender, *n* (%)	129 (69.0)
Mean Simplified Acute Physiology Score 3 score (SD)	59 (12)
Length of stay in intensive care unit, days, mean (range)	29 (2–145)
Previous non-antipseudomonal antibiotics, *n* (%)	79 (42.2)
*Antibiotics during ICU stay*	
Piperacillin/tazobactam, *n* (%)	114 (61.0)
Meropenem, *n* (%)	102 (54.8)
Ceftazidime, *n* (%)	73 (39.0)
Amikacin, *n* (%)	72 (38.5)
Ciprofloxacin, *n* (%)	69 (36.9)
*Types of antibiotics received*	
None	13 (7.4)
1	35 (19.9)
2–3	94 (53.5)
4–5	34 (19.2)
*Reason for ICU admission, n (%)*	
Cardiovascular/vascular	55 (29.4)
Respiratory	46 (24.6)
Neurological	32 (17.1)
Gastrointestinal	25 (13.4)
Sepsis/septic shock	15 (7.5)
Trauma	10 (5.3)
Others	5 (2.7)
Mechanical ventilation, *n* (%)	160 (83.8)
Therapy with one or more vasopressors, *n* (%)	169 (88.5)

*SD* standard deviation

During the ICU admission, 96% of the patients received at least one of the antipseudomonal antibiotics. The most common antibiotics used were piperacillin/tazobactam (61.0%) and meropenem (54.8%). The median length of stay was 29 days (range 2–145 days).

From these patients, 1158 cultures were available (mean number cultures per patient 6, range 2–42). At baseline, the number of patients with an isolate susceptible to the antibiotic was 176 (94.1%) for piperacillin/tazobactam, 171 (91.4%) for ceftazidime, 133 for ciprofloxacin (71.1%), 124 (66.3%) for meropenem and 155 (82.9%) for amikacin. During the follow-up, phenotype switch from susceptible/intermediate to resistant for at least one antibiotic occurred in isolates from 38.1% of the patients during the ICU stay. During the ICU stay, 31.6% of *Pseudomonas* isolates became resistant to piperacillin/tazobactam, 27.5% to ceftazidime, 22.0% to meropenem, 13.3% to ciprofloxacin and 12.9% to amikacin.

### Duration of antibiotics use and the emergence of resistance

Exposure to the relevant antibiotics was significantly associated with the emergence of resistance of *P. aeruginosa* for meropenem (*p* < 0.001), piperacillin/tazobactam (*p* = 0.006) and ceftazidime (*p* = 0.006). For ciprofloxacin, there was an association but did not reach statistic significant level (*p* = 0.06). There was no association with amikacin (*p* = 0.33). Other variables such as age, gender, SAPS 3 scores, previous antibiotic use, necessity use of mechanical ventilation or vasopressors and ICU length of stay were not associated with the emergence of *P. aeruginosa* resistance to any of the antipseudomonal antibiotics during ICU stay (data not shown).

Using antibiotics for less than 4 days was not significantly associated with emergence of resistance (Table 2). When the antibiotic was administered for longer than 15 days, we observed the emergence of resistance of *P. aeruginosa* to all antipseudomonal antibiotics, except to amikacin. The OR (95% CI) after adjusting for age, gender and SAPS 3 scores for the emergence of resistance for piperacillin–tazobactam was 4.7 (1.8–12.4) and for ciprofloxacin was 14.5 (2.8–75.0). Giving ceftazidime or meropenem between 8 and 15 days was associated with phenotypic switch of *P. aeruginosa* to these antibiotics. The ORs (95% CI) were 2.6 (1.1–6.0) and 10.0 (19.8–551.0), respectively. Further adjustment for length of stay changed the significant associations to not significant, except for meropenem (Table 2). The association between meropenem use and emergence of resistance remained even after adjustment for length of stay.

**Table 2:** Odds ratios for the emergence of antipseudomonal antibiotic resistance by *Pseudomonas aeruginosa* in relation to antibiotic duration

Antibiotic duration	Number of patients	Events (%)	Odds ratio (95% confidence interval) adjusted for age, gender and SAPS 3 scores	Odds ratio (95% confidence interval) adjusted for age, gender, SAPS 3 scores and length of stay
Piperacillin–tazobactam (*n* = 176)				
Reference (i.e., use of other antibiotic than piperacillin–tazobactam or no antibiotic)	65	13 (21.2)	1	1
1–3 days	17	6 (35.3)	2.5 (0.8–8.0)	2.3 (0.7–8.2)
4–7 days	29	11 (37.9)	2.3 (0.8–6.1)	2.2 (0.7–6.4)
8–15 days	34	9 (26.5)	1.5 (0.6–4.1)	1.5 (0.5–4.3)
>15 days	29	16 (55.2)	4.7 (1.8–12.4)^‡^	1.7 (0.6–5.3)
Ceftazidime (*n* = 171)				
Reference (i.e., use of other antibiotic than ceftazidime or no antibiotic)	99	22 (22.2)	1	1
1–3 days	13	0	NA	NA
4–7 days	12	8 (66.7)	1.7 (0.4–6.6)	1.7 (0.4–7.4)
8–15 days	34	14 (41.2)	2.6 (1.1–6.0)^‡^	1.7 (0.7–4.3)
>15 days	13	7 (53.8)	3.4 (1.0–12.4)^‡^	0.7 (0.2–3.4)
Ciprofloxacin (*n* = 128)				
Reference (i.e., use of other antibiotic than ciprofloxacin or no antibiotic)	78	7 (9)	1	1
1–3 days	18	2 (5.6)	1.5 (0.3–7.9)	2.0 (0.3–11.7)
4–7 days	10	0	0	0
8–15 days	13	3 (23.1)	2.7 (0.5–13.3)	1.4 (0.2–850)
>15 days	9	5 (55.6)	14.5 (2.8–75.0)^‡^	1.9 (0.2–15.6)
Meropenem (*n* = 123)				
Reference (i.e., use of other antibiotic than meropenem or no antibiotic)	64	3 (4.7)	1	1
1–3 days	13	0 (0)	0.0	0.0
4–7 days	9	2 (5.6)	4.9 (0.7–37.2)	3.4 (0.4–30.2)
8–15 days	21	17 (81.0)	104.5 (19.8–551.0)^‡^	79.1 (14.9–421.0)^‡^
>15 days	16	5 (31.2)	10.0 (2.0–49.5)^‡^	4.6 (1.7–32.2)^‡^
Amikacin (*n* = 148)				
Reference (i.e., use of other antibiotic than amikacin or no antibiotic)	87	10 (11.5)	1	1
1–3 days	41	7 (17.1)	1.3 (0.4–4.2)	0.7 (0.2–2.6)
4–7 days	13	1 (7.7)	0.3 (0.03–3.0)	0.1 (0.01–2.2)
8–15 days	7	1 (14.3)	0.5 (0.05–5.1)	0.1 (0.01–2.9)
>15 days	0	0	0.0	0.0

^‡^Statistically significant at *p* < 0.05

Among all antipseudomonal antibiotics, only meropenem use of 8–15 days and >15 days was associated with the emerging of resistance of any of antipseudomonal antibiotics. The ORs were 6.3 (2.4–16.8, *p* = 0.0001) and 2.6 (1.1–6.3, *p* = 0.04), respectively.

Eight patients (4.2%) were excluded due to possible cross-contamination. Re-analysis after excluding these patients did not significantly alter the results (data not shown).

### Association between the mode of administration of meropenem and combination beta-lactams and aminoglycosides with emergence of resistance

Extended administration of meropenem was not associated with a lower risk of emerging *P. aeruginosa* resistance in comparison with intermittent administration (OR 1.1 (0.2–4.0). The proportion of patients who acquired resistance was 45.5% for extended administration (*n* = 46) and 43.5% for intermittent administration (*n* = 11).

The combination of piperacillin/tazobactam with amikacin was also not associated with a lower risk of acquiring *P. aeruginosa* resistance [OR 1.0 (0.4–2.5)]. The proportion of patients who acquired resistance was 37.5% for combination therapy (*n* = 24) and 37.2% for patients with monotherapy (*n* = 86).

## Discussion

We observed in this study that exposure to meropenem, as short as 8 days, was already associated with emergence of resistance in *Pseudomonas aeruginosa*, even after adjustment for length of stay. We also found that extended infusion of meropenem and combination of beta-lactam antibiotics with amikacin did not reduce emergence of resistance. The present study used patient-based instead of surveillance-based approach. Instead of detecting the emergence of resistance in patients who endured regular screening cultures, the emergence of resistance was studied in clinical cultures. This approach is convenient in a clinical situation where surveillance cultures are not always routinely performed. The results of the present study are in line with those from surveillance-based approach studies that showed carbapenem as antibiotic class with the strongest potential for selection of resistance, for example from Carmeli et al. [15]. Unlike Carmeli and co-workers who used imipenem in their study, we used meropenem. Whether one type of carbapenem is more associated with emergence of resistance should be further explored. Earlier studies also showed that antipseudomonal antibiotics piperacillin/tazobactam, ceftazidime and ciprofloxacin were associated with the emergence of *P. aeruginosa* resistance in critically ill patients [7, 16, 17]. However, in our study, the significant effect disappeared after further adjustment with the length of stay, except for meropenem, which highlights meropenem as an antibiotic that selected for *P. aeruginosa* resistance.

Several studies have been published recently that investigated the molecular mechanisms of the resistance of *P. aeruginosa* to antipseudomonal antibiotics showing that mutated genes during the emergence of beta-lactam resistance differed for each antibiotic [18, 19]. Since we do not perform molecular analysis, our study does not allow the analysis of mechanisms involved in the emergence of resistance, either expression of intrinsic resistance mechanisms in response to antimicrobial exposure such as reduction in expression of the outer membrane proteins (porins) or increase production of an efflux pump to a variety of antibiotics.

To the best of our knowledge, the present study is the first to show that the emergence of resistance in initially susceptible *P. aeruginosa* isolates does not occur when the antibiotic was given for less than 4 days, even for meropenem. This is in line with results from Lodise et al. [6] that showed patients infected with a carbapenem-resistant *P. aeruginosa* were more likely to have been exposed to carbapenems for 3 days or more compared to patients infected with a carbapenem susceptible strain. The combination therapy beta-lactam and amikacin failed to decrease the emergence of resistance unlike in an in vitro study [20]. One of the possible explanations is that amikacin drug concentration may be suboptimal for *P. aeruginosa* in ICU patients. We did not always perform peak measurement of amikacin, and therefore, we do not know whether the pharmacokinetic/pharmacodynamic (PK/PD) target was achieved. In this study, the dose of 15 mg/kg divided into three doses was mostly used, yet some authors advised the use of 25 mg/kg/day [21].

Extended meropenem infusion was also not associated with less emergence of resistance compared with intermittent infusion. This effect may be true but alternative explanations such as optimal dosing are also possible and further study on this issue is needed.

This study has further limitations. Firstly, although using clinical specimens approach is convenient and mimic daily practice in many ICUs, this study cannot determine the exact date of phenotype switch because cultures were not regularly performed. Since the study was based on clinical specimens, the follow-up duration of the patients varied, i.e., the follow-up duration was the time frame between the first and the last positive culture for *P. aeruginosa*. A prospective study design with cultures taken at regular time frame will be a better study design despite in the routine practice *P. aeruginosa* could not always be re-isolated. Secondly, this study does not use genotyping to investigate the relatedness of the strains and to confirm the cross-transmission of resistant *P. aeruginosa*. However, cross-transmission estimated in this study (4.2%) is comparable with cross-transmission confirmed by genotyping (2.8%) as shown in a previous study [7], and the results of the analysis remain the same after excluding possible cross-transmission. It is also possible that several strains could be responsible for infection and this selection pressure cannot be differentiated from the emergence of resistance since no genotyping was performed. Thirdly, the results of this study might underestimate the effect since no minimum inhibitory concentration (MIC) values were available. Antipseudomonal antibiotics use might be associated with increasing MIC values that does not cause phenotypic switch on disk diffusion. Fourthly, the number of patients was relatively small and the study may be underpowered for several antibiotics that were used in low number, and lastly this study was performed in one center only.

## Conclusions

The results of this study suggested that meropenem is associated with resistance of *P. aeruginosa* as soon after 8 days of use. This finding should be confirmed in other studies. All antipseudomonal antibiotics except amikacin are associated with the emergence of *P. aeruginosa* resistance. Physicians should weigh up the risk of resistance and the therapeutic benefit when meropenem or other antipseudomonal antibiotics are going to be used for a long period. Moreover, when physicians start empiric therapy with a carbapenem antibiotic, they should consider to de-escalate it within 48–72 h, once the culture results are known since the switch might be clinically beneficial for the patients.

## Abbreviations

ICU: intensive care unit
SAPS 3: Simplified Acute Physiology Score 3
CLSI: Clinical & Laboratory Standards Institute
SD: standard deviation
OR: odds ratio
95% CI: 95% confidence interval
MIC: minimum inhibitory concentration
